# Case Report: Extracorporeal Carbon Dioxide Removal in a Severely Brain‐Injured Patient With Acute Respiratory Distress Syndrome

**DOI:** 10.1155/crcc/5404072

**Published:** 2025-12-19

**Authors:** Sébastien Gibot, Lionel Nace, Aurélie Cravoisy

**Affiliations:** ^1^ Service de Médecine Intensive et Réanimation-Hôpital Central, CHRU de Nancy, Nancy, France

**Keywords:** acute respiratory distress syndrome, extracorporeal CO_2_ removal, intracranial pressure, subarachnoid haemorrhage

## Abstract

The management of acute respiratory distress syndrome (ARDS) in a patient suffering from a severe brain injury may be difficult, especially when hypercapnia occurs. The rise of PaCO_2_ may compromise cerebral hemodynamics and increase intracerebral pressure (ICP). We describe herein a case of a 41‐year‐old man with a severe subarachnoid haemorrhage who develops a severe ARDS consequent to an inhalation pneumonia. Despite optimisation of mechanical ventilation, respiratory mechanics worsened and led to a major hypercapnic acidosis associated with an ICP rise. Because of concomitant ICP elevation and hemodynamic instability, prone positioning was considered too high‐risk. We therefore opted for an extracorporeal carbon dioxide removal (ECCO_2_R) technique first. PaCO_2_ rapidly decreased, as well as ICP, and the patient could finally be proned while in ECCO_2_R. ECCO_2_R was kept for a total of 77 h with no complications. Thereafter, the patient progressively improved and could be weaned from the ventilator after 27 days. He was evaluated 3 months later during an outpatient visit. He was doing well with no sequelae and has resumed previous activities. While the use of extracorporeal decarboxylation techniques is being studied in ARDS, specific investigation in severely brain‐injured patients deserves to be conducted.

## 1. Introduction

Lung protective ventilation is a recognised strategy for the treatment of acute respiratory distress syndrome (ARDS) [1]. Although this strategy may lead to or favour hypercapnia due to the tidal volume lowering, this can be well tolerated in the vast majority of patients. However, in the case of cerebral aggression, hypercapnia may jeopardise cerebral hemodynamics and autoregulation, resulting in intracranial pressure (ICP) increase [2]. Therefore, the treatment of patients suffering from concomitant cerebral lesions and ARDS may be challenging.

We describe the case of a young patient presenting with both severe subarachnoid haemorrhage (SAH) and ARDS. Severe respiratory acidosis developed and was treated by extracorporeal CO_2_ removal.

## 2. Case Report

### 2.1. Presentation

A 41year‐old male patient was admitted to this intensive care unit (ICU) because of a SAH.

The patient had been in his usual state of health when he suddenly presented an episode of agitation for which he was taken to the emergency department of another hospital. His medical history was only remarkable for chronic drug abuse.

On examination, the temporal temperature was 36.7°C, the blood pressure 134/87 mmHg, the pulse 72 bpm, the respiratory rate 18 breaths per minute, and the oxygen saturation 94% while the patient was breathing room air. He again presented an episode of agitation, associated with dysarthria and diaphoresis. Pupils were in symmetric reactive myosis.

The white blood cells count was 32,370 per microliter, and blood glucose was 15.07 mmol/L. There were no other abnormalities. Urinary drug screening was positive for benzodiazepine.

As agitation became extreme, 20 mg diazepam IV was administered, but with no effect. General anaesthesia was therefore performed (etomidate and suxamethonium) before intubation and the start of mechanical ventilation under deep sedation with midazolam and sufentanil. Severe arterial hypertension occurred (225/117 mmHg) for which IV nicardipine was administered.

**Figure 1:**
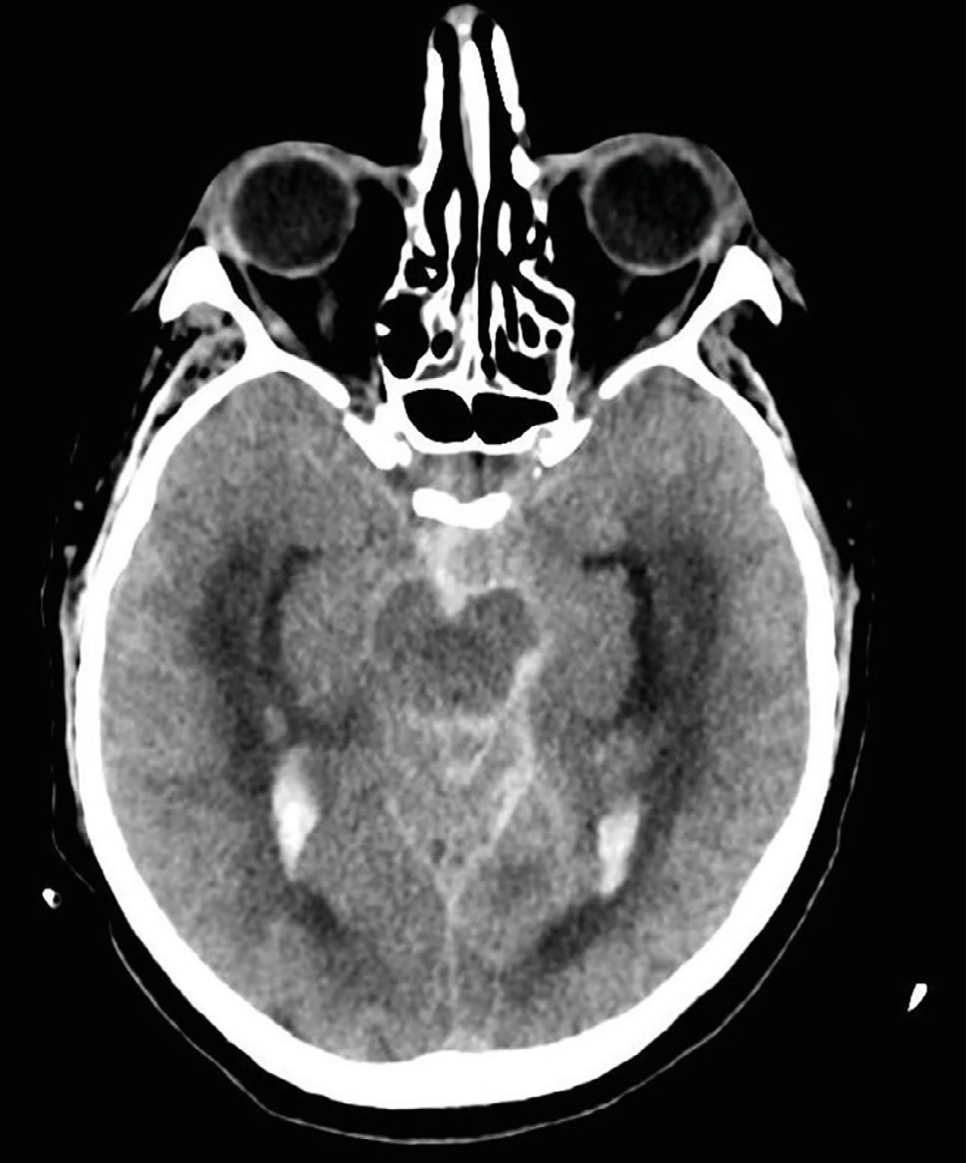
Head CT scan on admission.

Chest X‐ray was normal. Head CT scan (Figure 1) revealed the presence of a SAH Fischer grade 4 (Fischer scale ranging from 1, the less severe, to 4) and a left internal carotid siphon aneurysm.

The patient was referred to the neuroradiology department of this hospital on the same day: the internal carotid aneurysm was secured with four coils, an external ventricular drain (EVD) was placed, as well as an ICP monitor. The patient was next admitted to this ICU.

On admission, body temperature was 38.2°C, arterial blood pressure 127/62 mmHg, the pulse rate 82 bpm, the respiratory rate 24 breaths per minute, and the oxygen saturation 96% under invasive protective mechanical ventilation. Tracheal secretions were purulent. Pupils were in symmetric reactive myosis, and the EVD was permeable and placed at + 15 cmH_2_O. The ICP was 15 cmH_2_O.

The arterial pH was 7.36, PaCO_2_ 39 mmHg, PaO_2_/FIO_2_ 163. There were no biological abnormalities.

Sedation with midazolam and sufentanil was continued, nimodipine was administered IV, and amoxicillin/clavulanate was started because of high suspicion of inhalation pneumonia. The head was elevated at 30°, and external cooling started.

Cerebral perfusion pressure (CPP) was maintained around 65–70 mmHg. Transcranial Doppler was iteratively used to detect vasospasm.

The next day, a scheduled head CT scan was performed and showed a partial regression of the SAH, though with the appearance of small left cerebellar ischemic defects.

Treatment remained unchanged in wait for the next scheduled head CT scan.

Respiratory mechanics progressively worsened despite protective lung ventilation and early antibiotics because of the occurrence of ARDS complicating the inhalation pneumonia: PaO_2_/FIO_2_ 82, respiratory static compliance (C_rs_) 23 mL/cmH_2_O.

**Figure 2:**
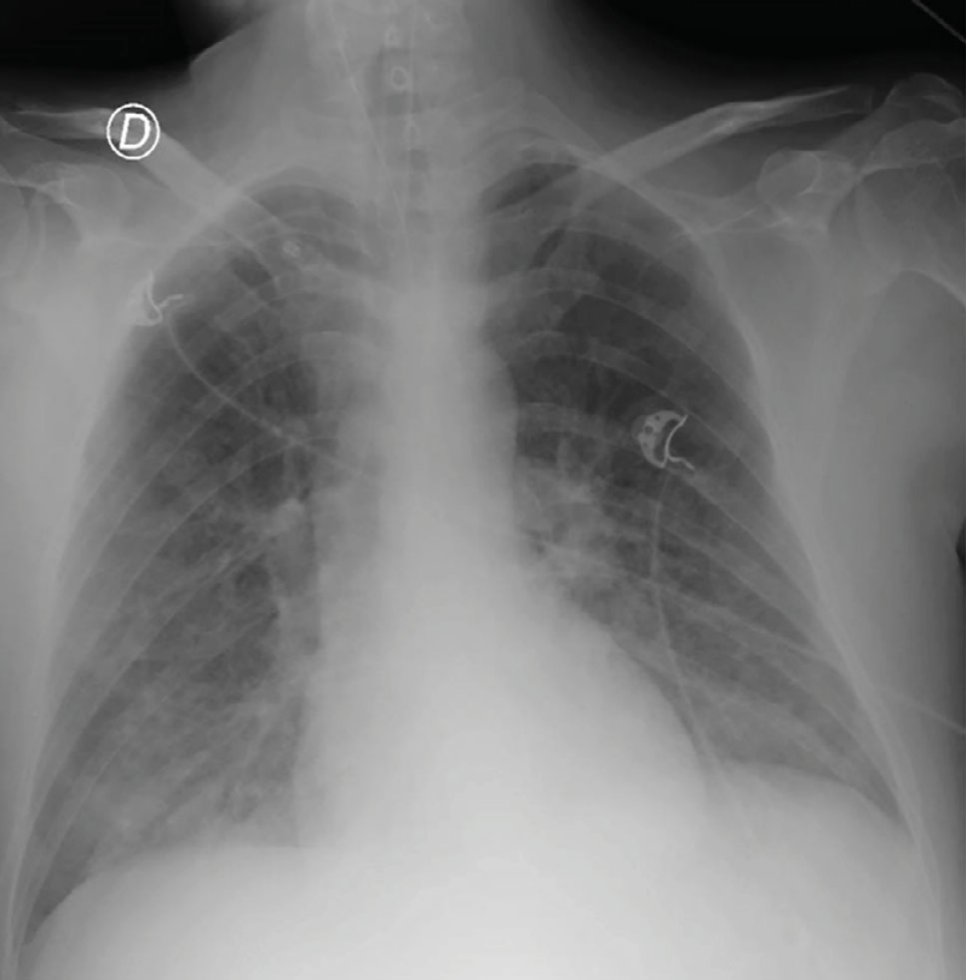
Chest X‐ray performed at day 3.

Chest X‐ray revealed bilateral alveolar opacities (Figure 2). The arterial pH was 7.33, and PaCO_2_ was 50 mmHg. At this time, hemodynamic instability occurred, and norepinephrine was started. ICP remained around 16 cmH_2_O, and repeated TCDs were reassuring, although CPP was hard to keep above 65 mmHg. In this situation of hemodynamic instability and raised ICP, we considered a trial of prone positioning at too high‐risk and opted for an optimization of the mechanical ventilation under paralysis with cisatracurium.

Unfortunately, a severe respiratory acidosis developed, associated with a further increase in ICP and the persistence of hemodynamic compromise. Respiratory parameters were as follows: respiratory rate 30/min, tidal volume 500 mL (6.1 mL/kg ideal body weight), positive end‐expiratory pressure (PEEP) 3 cmH_2_O, plateau pressure (P_plat_) 36 cmH2O, peak inspiratory pressure (P_peak_) 61 cmH2O, C_rs_ 15 mL/cmH_2_O; PaO_2_ 77 mmHg, PaCO_2_ 86 mmHg and pH 7.07 under FIO_2_ 1. Of note, a small increase of PEEP led to a disproportionate increase of P_plat_ and driving pressure. Therefore, PEEP was kept at 3 cmH_2_O.

## 3. Management

**Figure 3:**
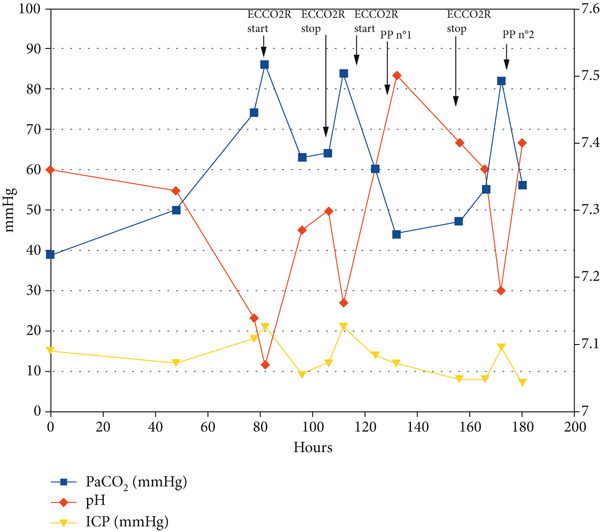
Arterial blood gas and intracranial pressure evolution.

To manage this severe hypercapnia, extracorporeal carbon dioxide removal (ECCO_2_R) was envisioned. A 12‐French double‐lumen catheter was placed in the right internal jugular vein, and ECCO_2_R was started using the PrismaLung+ membrane on the PrisMax 2 system (Baxter, Illinois, United States). Because of the intracranial haemorrhage, the full anticoagulation required to avoid membrane clotting could not be administered. In order to preserve the blood‐gas exchanger, we therefore coupled ECCO_2_R to continuous renal replacement therapy (CRRT) using the ST150 hemofiltration membrane that allowed blood predilution (Baxter). Settings were as follows: blood flow 320 mL/min, predilution 500 mL/h, postdilution 500 mL/h, sweep gas (oxygen) 9 L/min. Careful anticoagulation was achieved with continuous IV unfractionated heparin 10000 IU/24H: anti‐Xa was kept at 0.2 IU/mL. Under this treatment, PaCO_2_ rapidly dropped from 86 mmHg to 65 mmHg, and arterial pH increased from 7.07 to 7.23 (Figure 3). After 24H, the transmembrane pressure increased, and the therapy had to be stopped. Severe hypercapnia occurred again, and ECCO_2_R was resumed again, associated with an improvement of the respiratory acidosis. Twelve hours later, hemodynamics were stable without vasopressors, and ICP remained below 12 cmH_2_O. As the patient was still hypoxemic (PaO_2_/FIO_2_ 94), he was prone positioned (PP) for 16 h: hypoxemia sharply improved, and PaCO_2_ further decreased from 60 mmHg to 43 mmHg. ECCO_2_R was maintained for an additional 18 h after the patient had returned to a supine position (total duration of ECCO_2_R: 24 + 53 = 77 h). At this time, respiratory parameters were as follows: respiratory rate 25/min, tidal volume 500 mL (6.1 mL/kg ideal body weight), PEEP 6 cmH_2_O, P_plat_ 31 cmH2O, P_peak_ 52 cmH2O, C_rs_ 20 mL/cmH_2_O; PaO_2_ 71 mmHg, PaCO_2_ 48 mmHg and pH 7.46 under FIO_2_ 0.35. A second PP was performed for 16 h because of the recurrence of hypoxemia and respiratory acidosis, again with a very good response. Thereafter, lung function continuously improved, despite the occurrence of ventilator‐associated pneumonia (*Klebsiella pneumoniae*).

## 4. Follow‐Up

Repeated head CT scans were satisfactory with a progressive regression of intracranial haemorrhage and cerebellar ischaemia, and a normal ventricular volume: EVD was withdrawn after 17 days. The patient was progressively weaned from the ventilator and finally extubated after 27 days. Neurologic exam revealed no abnormalities. After another 6 days, the patient was breathing room air and was able to mobilise and eat by himself. He was discharged to the neurologic ward and finally to home after a week. He was evaluated 3 months later during an outpatient visit by the ICU team. He was doing well with no physical, cognitive or psychological sequelae and has resumed previous activities.

## 5. Discussion

The management of ARDS in patients with brain injury may be difficult when hypercapnia occurs. When PaCO_2_ increases, cerebral autoregulation is impaired, especially when PaCO_2_ becomes higher than 70 mmHg [2]. Although optimization of mechanical ventilation and prone positioning are often effective, some patients remain severely hypercapnic. Moreover, as in the case we describe, prone positioning may not be safely feasible because of hemodynamic instability and raised ICP.

Several extracorporeal techniques have been developed to achieve decarboxylation, either arteriovenous (e.g., iLA membrane ventilator, Novalung, Germany), venovenous (e.g., Hemolung, ALung, United States) or using CRRT machines (e.g., PrismaLung, Baxter, United States). These devices have been extensively evaluated for the treatment of ARDS or COPD patients, but scarcely in brain‐injured patients. In 10 severe traumatic brain‐injured patients with moderate ARDS, Munoz‐Bendix et al. showed that low‐flow, pumpless, arteriovenous extracorporeal decarboxylation was feasible and enabled lung‐protective ventilation [3]. However, in this report, pretreatment PaCO_2_ was not that high (46.6 mmHg) and only decreased to 39.7 mmHg. Nevertheless, this slight reduction of PaCO_2_ was sufficient to control raised ICP.

Veno‐venous extracorporeal membrane oxygenation (VV‐ECMO) allows for both oxygenation and decarboxylation and can be implemented in cases of refractory severe ARDS [4]. However, vigorous anticoagulation is mandatory to prevent circuit clotting. The Extracorporeal Life Support Organization (ELSO) guidelines recommend unfractionated heparin: a 50–100 units/kg bolus followed by a continuous infusion of 20–50 units/kg/h in order to achieve an activated clotting time of 180–220 s. In the recent cohort study, Hwang et al. observed that 80% of deceased patients under ECMO presented with lobar cerebral microbleeds or SAH [5]. In the EOLIA study, 27% of patients under VV‐ECMO developed severe thrombocytopenia (< 20 G/L), and 47% developed severe bleeding [6]. We therefore considered this technique too high‐risk for our patient.

Recommendations for anticoagulation during ECCO_2_R have recently been published, and the use of unfractionated heparin to target an anti‐Xa activity between 0.3 and 0.5 IU/mL is proposed [7]. However, it still carries a risk of bleeding, as shown by McNamee et al. in the REST trial, with 4.5% of patients developing an intracranial haemorrhage [8]. For these reasons, we here choose to reduce anticoagulation, keeping anti‐Xa activity at 0.2 IU/mL and add a 500 mL/h blood predilution.

To the best of our knowledge, this is the first time that the use of a carbon dioxide removal with a continuous renal replacement device in a brain‐injured patient with severe ARDS is reported. This technique was effective in rapidly decreasing life‐threatening hypercapnia. Because recommended anticoagulation was considered at too high risk in our patient, we used minimal unfractionated heparin coupled with hemofiltration to limit membrane clotting: with this strategy, the membrane lifespan remained acceptable (24 h for the first and 53 h for the second membrane). Although ECCO_2_R had no effect on oxygenation, it achieved the control of respiratory acidosis leading to a decrease of ICP and hemodynamic stabilization and allowed us to safely prone the patient.

## 6. Conclusion

We report the case of a severely brain‐injured patient who develops a secondary ARDS responsible for an intractable hypercapnia jeopardising cerebral perfusion successfully treated by extracorporeal CO_2_ removal.

While the use of extracorporeal decarboxylation techniques is being studied in ARDS, there is still a lack of evidence regarding outcomes, with concerns of high rates of adverse events [9]. However, specific investigation in severely brain‐injured patients deserves to be conducted.

## Ethics Statement

Written informed consent was obtained from the patient for the publication of this case report.

## Conflicts of Interest

The authors declare that the research was conducted in the absence of any commercial or financial relationships that could be construed as a potential conflict of interest.

## Author Contributions

Sébastien Gibot: conceptualisation, writing the original draft. Lionel Nace: conceptualisation, reviewed and edited the manuscript. Aurélie Cravoisy: conceptualisation, reviewed and edited the manuscript.

## Funding

No funding was received for this manuscript.

## Data Availability

All pertinent data are included in the article. Further inquiries can be directed to the corresponding author.
